# β-apo-10′-carotenoids support normal embryonic development during vitamin A deficiency

**DOI:** 10.1038/s41598-018-27071-3

**Published:** 2018-06-11

**Authors:** Elizabeth Spiegler, Youn-Kyung Kim, Beatrice Hoyos, Sureshbabu Narayanasamy, Hongfeng Jiang, Nicole Savio, Robert W. Curley, Earl H. Harrison, Ulrich Hammerling, Loredana Quadro

**Affiliations:** 1 Department of Food Science, Rutgers Center for Lipid Research, and New Jersey Institute for Food, Nutrition, and Health, Rutgers University, New Brunswick, New Jersey 08901 United States; 2 Immunology Program, Memorial Sloan-Kettering Cancer Center, New York, New York 10065 United States; 3 College of Pharmacy, The Ohio State University, Columbus, OH 43210 United States; 4 Department of Human Sciences, The Ohio State University, Columbus, OH 43210 United States; 5 College of Physicians and Surgeons, Department of Medicine, Columbia University, New York, NY 10032 United States

## Abstract

Vitamin A deficiency is still a public health concern affecting millions of pregnant women and children. Retinoic acid, the active form of vitamin A, is critical for proper mammalian embryonic development. Embryos can generate retinoic acid from maternal circulating β-carotene upon oxidation of retinaldehyde produced *via* the symmetric cleavage enzyme β-carotene 15,15′-oxygenase (BCO1). Another cleavage enzyme, β-carotene 9′,10′-oxygenase (BCO2), asymmetrically cleaves β-carotene in adult tissues to prevent its mitochondrial toxicity, generating β-apo-10′-carotenal, which can be converted to retinoids (vitamin A and its metabolites) by BCO1. However, the role of BCO2 during mammalian embryogenesis is unknown. We found that mice lacking BCO2 on a vitamin A deficiency-susceptible genetic background (*Rbp4*^−/−^) generated severely malformed vitamin A-deficient embryos. Maternal β-carotene supplementation impaired fertility and did not restore normal embryonic development in the *Bco2*^−/−^*Rbp4*^−/−^ mice, despite the expression of BCO1. These data demonstrate that BCO2 prevents β-carotene toxicity during embryogenesis under severe vitamin A deficiency. In contrast, β-apo-10′-carotenal dose-dependently restored normal embryonic development in *Bco2*^−/−^*Rbp4*^−/−^ but not *Bco1*^−/−^*Bco2*^−/−^*Rbp4*^−/−^ mice, suggesting that β-apo-10′-carotenal facilitates embryogenesis as a substrate for BCO1-catalyzed retinoid formation. These findings provide a proof of principle for the important role of BCO2 in embryonic development and invite consideration of β-apo-10′-carotenal as a nutritional supplement to sustain normal embryonic development in vitamin A-deprived pregnant women.

## Introduction

Mammalian embryonic development requires vitamin A for normal cell fate specification, patterning and differentiation^1^. These activities are attributed predominantly to retinoic acid, the vitamin A metabolite that binds the nuclear retinoic acid receptors to regulate the transcription of hundreds of genes that are critical for normal embryogenesis^2,3^. Both gestational vitamin A deficiency and excess can cause gross morphological defects in the eyes, limbs, heart, nervous system and craniofacial region^1,4^. Vitamin A deficiency affects hundreds of millions of children and pregnant women in developing countries^5,6^, and is prevalent in populations reliant on β-carotene, the most abundant dietary provitamin A source of retinoids (vitamin A and its derivatives)^7,8^.

The generation of retinoids from β-carotene requires enzymatic cleavage, producing apocarotenoids that in turn can be converted to retinoic acid. Symmetric β-carotene cleavage to retinaldehyde by the cytosolic enzyme β,β-carotene 15,15′-oxygenase (BCO1) is thought to be the main pathway for retinoid generation from carotenoids in both adult^9,10^ and embryonic tissues^11^. Human vitamin A deficiency is well-modeled by mice lacking retinol-binding protein (RBP4 or RBP), the sole specific carrier for retinol in the bloodstream^12^. On the vitamin A deficiency-susceptible *Rbp4*^−/−^ background, *Bco1*^−/−^*Rbp4*^−/−^ female mice generated severely malformed *Bco1*^+/−^*Rbp4*^−/−^ embryos (carrying one copy of the wild-type *Bco1* allele), whereas β-carotene supplementation rescued 61% of these offspring from developmental defects, since the embryos could cleave β-carotene *via* BCO1^11^.

On the other hand, asymmetric β-carotene cleavage by the mitochondrial enzyme β,β-carotene 9′,10′-oxygenase (BCO2) generates β-ionone and β-apo-10′-carotenal^13^. The latter can be cleaved by BCO1, yielding retinaldehyde and its downstream derivatives, retinol and retinoic acid^10,14^. Nevertheless, this asymmetric cleavage pathway is not considered a major source of retinoids, but rather a pathway to reduce oxidative stress resulting from the toxic accumulation of carotenoids in mitochondria^15,16^. A few intriguing reports have indicated that apocarotenoids can restore growth in vitamin A-deprived rats, chickens and quails^17–19^; however, this potentially critical biological activity has never gained full acceptance, nor has it been unequivocally established whether apocarotenoids perform this action *per se* or upon conversion to retinoids.

Here, we explore the function of BCO2 and its cleavage products in developing mammalian embryos. We demonstrate that BCO2 is critical for preventing β-carotene toxicity during embryogenesis under severe vitamin A deficiency, and that β-apo-10′-carotenal can restore normal development in a mouse model of severe vitamin A deficiency. These findings provide key insights into the unsolved problem of gestational vitamin A deficiency.

## Results

### Lack of BCO2 exacerbates embryonic vitamin A deficiency

**Figure 1:**
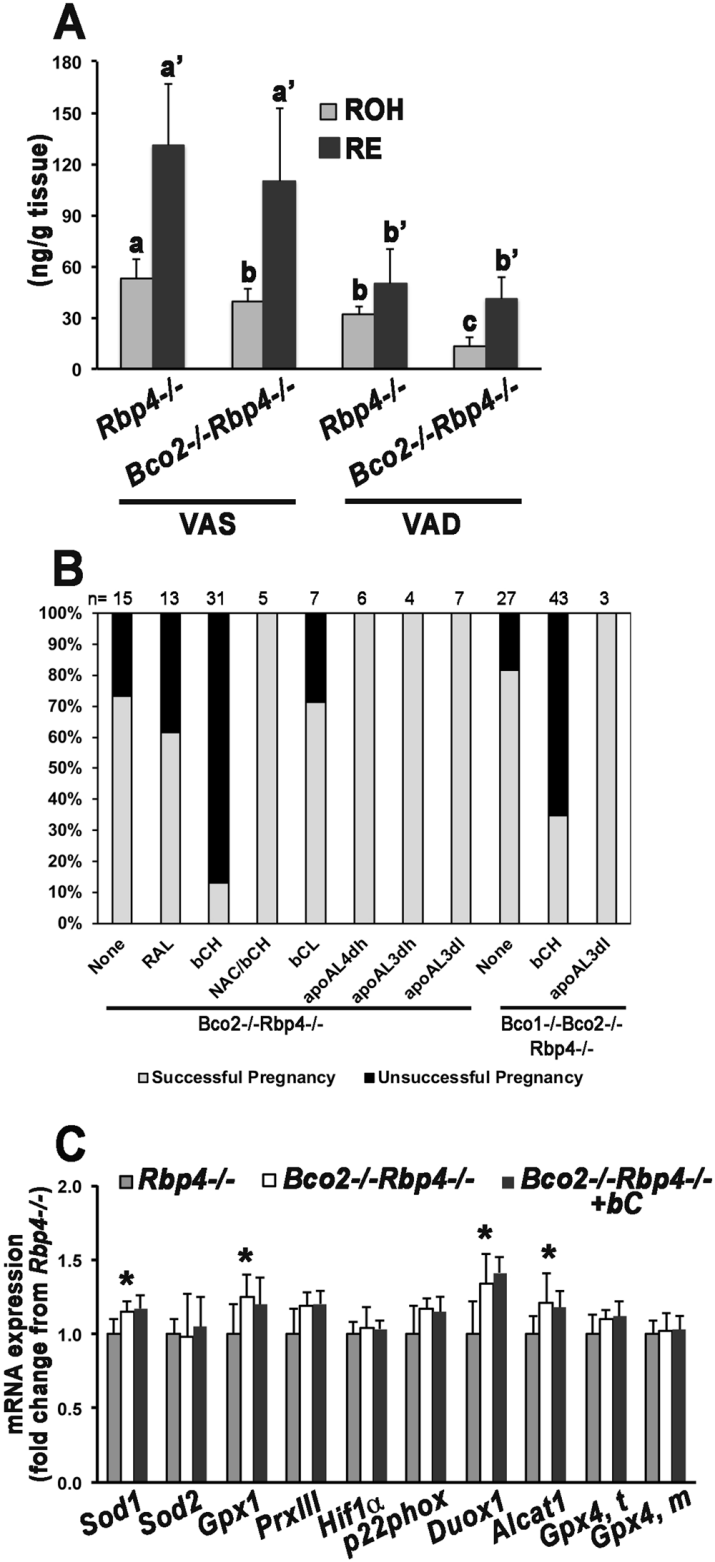
BCO2 regulates embryonic retinoid levels, β-carotene toxicity and oxidative stress. (**A**) HPLC analysis of retinol (ROH) and retinyl esters (RE) in *Rbp4*^−/−^ and *Bco2*^−/−^*Rbp4*^−/−^ 14.5-dpc embryos from dams of the same genotype fed a VAS or VAD diet from 0.5 dpc. n = 6–10 embryos/group. Data are means ± SDs. Labeled means (within each metabolite) without a common letter differ, p < 0.05. (**B**) The percentage of unsuccessful pregnancies for each group was calculated as the ratio between the number of pregnant females at 14.5 dpc and the total number of females in which a vaginal plug was detected in that group. The X-axis displays the genotype and regimen of maternal supplementation: None, no supplementation; RAL, retinaldehyde, 1600 μg/day in diet from 7.5–9.5 dpc; bC^H^, β-carotene high dose (800 μg/day by IP injection from 6.5–9.5 dpc); NAC/bC^H^, 0.5 mg of N-acetylcysteine/g bodyweight from 0.5 dpc to 14.5 dpc in the water, and IP injection of bC^H^; bC^L^, β-carotene low dose (175 μg/day by IP injection from 6.5–9.5 dpc); apoAL^4dh^, β-apo-10′-carotenal 800 μg/day by IP injection from 6.5–9.5 dpc; apoAL^3dh^, β-apo-10′-carotenal 800 μg/day from 7.5–9.5 dpc either in the food or by IP injection; apoAL^3dl^, β-apo-10′-carotenal 400 μg/day from 7.5–9.5 dpc either in the food or by IP injection. n, total number of females in which a vaginal plug was detected. (**C**) qPCR analysis of oxidative stress response genes in 14.5-dpc embryos from *Rbp4*^−/−^ and *Bco2*^−/−^*Rbp4*^−/−^ dams fed the vitamin A-deficient diet from 0.5 dpc, and from *Bco2*^−/−^*Rbp4*^−/−^ dams fed the vitamin A-deficient diet from 0.5 dpc and administered 800 μg of β-carotene (bC)/day by IP injection from 6.5–9.5 dpc. *Rbp4*^−/−^ embryos were set as the calibrator at 1. Data are means ± SDs fold of *Rbp4*^−/−^; n = 4–8 embryos/group (from 3–4 dams/group). Statistical analysis performed by Student’s *t* test or the Mann-Whitney U test to compare embryos from non-injected *Rbp4*^−/−^ vs. *Bco2*^−/−^*Rbp4*^−/−^, and embryos from injected vs. non-injected *Bco2*^−/−^*Rbp4*^−/−^. *p < 0.05 vs. *Rbp4*^−/−^. *Gpx4*, *t*: total Gpx4; *Gpx4*, *m*: mitochondrial isoform of Gpx4.

**Figure 2:**
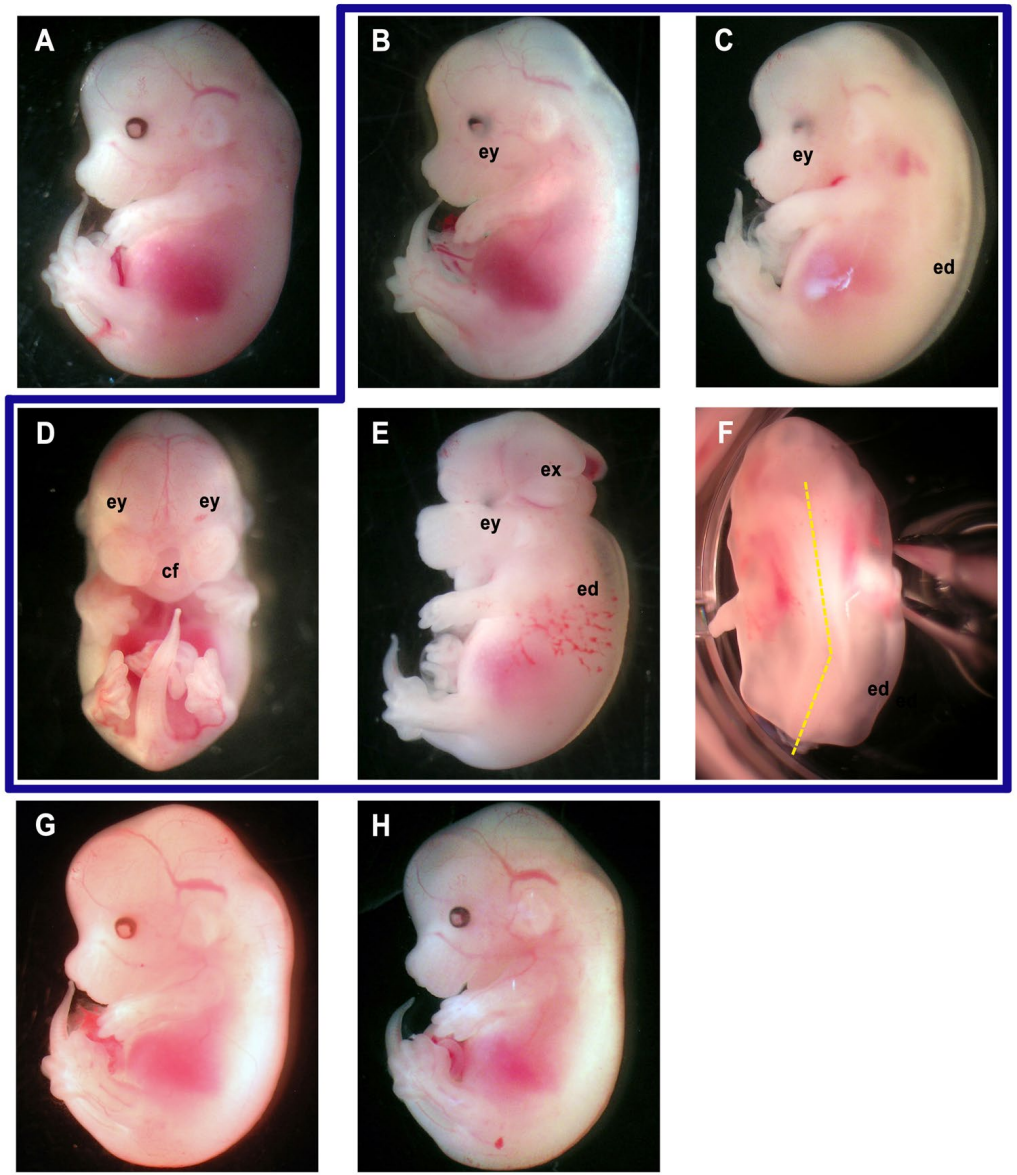
Gross morphology of embryos from *Bco2*^−/−^*Rbp4*^−/−^ dams under various regimens of dietary vitamin A intake and supplementation during pregnancy. *Bco2*^−/−^*Rbp4*^−/−^ embryos were collected at 14.5 dpc from *Bco2*^−/−^*Rbp4*^−/−^ dams on the following regimens: (**A**) vitamin A-sufficient diet (18 IU vitamin A/g and < 1.2 μg/g β-carotene). (**B**–**F**) Vitamin A-deficient diet (<0.2 IU/g and 0 μg/g β-carotene or other carotenoids); representative pictures are shown of phenotypes observed. Congenital defects marked as follows: ey, abnormal eye; ed, peripheral edema; cf, abnormal mid-facial region (snout foreshortened and divided by a sagittal median cleft, prolabium absent, maxillary process bearing whiskers separated by a larger-than-normal distance); ex, exencephaly; yellow dotted lines along the dorsal spine demonstrate the twist of the spine. (**G**) vitamin A-deficient diet and supplemented with retinaldehyde (1600 μg/day in the diet, 7.5–9.5 dpc). (**H**) vitamin A-deficient diet and supplemented with β-apo-10′-carotenal (800 μg/day by IP injection from 6.5–9.5 dpc). The same magnification was used for all panels.

To investigate the role of BCO2 during mammalian embryogenesis, we generated *Bco2*^−/−^*Rbp4*^−/−^ mice, which were prone to vitamin A deficiency due to the absence of RBP4^12^. *Bco2*^−/−^*Rbp4*^−/−^ mice were viable and fertile when maintained on a vitamin A-sufficient (VAS) chow diet. Although the embryonic retinol levels were lower in *Bco2*^−/−^*Rbp4*^−/−^ mice than in *Rbp4*^−/−^ mice (Fig. 1A), *Bco2*^−/−^*Rbp4*^−/−^ mice generated normal embryos on a VAS diet (Fig. 2A).

When *Bco2*^−/−^*Rbp4*^−/−^ dams were fed a purified vitamin A-deficient (VAD) diet during pregnancy, their vitamin A status became more tenuous (Table 1), and their embryonic retinol levels were further reduced compared to the *Rbp4*^−/−^ (Fig. 1A) and to wild-type (WT) embryos from WT dams under the same dietary conditions (Supplementary Fig. S1). Correspondingly, all the *Bco2*^−/−^*Rbp4*^−/−^ embryos from vitamin A-deprived dams were grossly malformed (Table 2a), and these external malformations were more severe than those shown by *Rbp4*^−/−^ embryos from vitamin A-deprived *Rbp4*^−/−^ dams (100% of the *Rbp4*^−/−^ embryos exhibited both eye defects and peripheral edemas^12^). Indeed, although 100% of the *Bco2*^−/−^*Rbp4*^−/−^ embryos displayed malformed eyes and peripheral edemas (Fig. 2B,C), 76% of them also presented an abnormal mid-facial region (cleft face and palate; Fig. 2D). To confirm the vitamin A-dependence of this embryonic phenotype, we supplemented the VAD diet of the *Bco2*^−/−^*Rbp4*^−/−^ dams with retinaldehyde (1600 μg/day, 7.5–9.5 days *post coitum* [dpc]^20^). Indeed, retinaldehyde treatment allowed 72% of the embryos to develop normally (Table 2b and Fig. 2G). Overall, using the well-studied *Rbp4*^−/−^ mouse model of vitamin A deficiency, we found that BCO2 deficiency exacerbated embryonic retinol deficiency and the associated malformations.

**Table 1:** Serum and hepatic retinol and retinyl ester concentrations of *Bco2*^−/−^*Rbp4*^−/−^ dams.

n	Serum (μg/dL)		n	Liver (μg/g)	
	Retinol	Retinyl Ester		Retinol	Retinyl Ester
*Vitamin A*-*sufficient diet*					
4	1.76 ± 0.18	2.51 (0–3.82)	4	2.40 ± 0.24	307 ± 114
*Vitamin A*-*deficient diet*					
7	1.16 ± 0.38*	1.26 (0–5.34)	7	2.29 ± 0.49	212 ± 81

Dams were fed a vitamin A-sufficient chow diet (18 IU vitamin A/g and <1.2 μg/g β-carotene) or a purified vitamin A-deficient diet (<0.2 IU/g and 0 μg/g β-carotene or other carotenoids) throughout pregnancy, and their tissues were collected at 14.5 dpc for HPLC analysis. Data are means ± SDs, except for serum retinyl ester [geometric mean (range)]. Statistical analysis was performed by Student’s *t* test or the Mann-Whi*t*ney U test as appropriate. *p < 0.05 vs. vitamin A-sufficient diet.

**Table 2:** Phenotype distribution of *Bco2*^−/−^*Rbp4*^−/−^ embryos at 14.5 dpc from dams fed the vitamin A-deficient diet during pregnancy and under various regimens of maternal supplementation.

Supplem.	n [embryos (dams)]	Normal	Malformed
a. None	72 (11)	0%	100%
b. RAL	36 (8)	72%	28%
c. bC^H^	30 (4)	3%	97%
d. NAC/bC^H^	35 (5)	3%	97%
e. bC^L^	31 (7)	0%	100%
f. apoAL^4dh^	36 (6)	77%	23%
g. apoAL^3dh^	25 (4)	40%	60%
h. apoAL^3dl^	36 (7)	25%	75%

Females mated with males of the same genotype. Embryonic genotype as maternal genotype. Supplem., regimen of maternal supplementation; None, no supplementation; RAL, retinaldehyde, 1600 μg/day in diet from 7.5–9.5 dpc; β-carotene (bC); bC^H^, high dose, 800 μg/day by IP injection from 6.5–9.5 dpc; NAC/bC^H^, 0.5 mg of N-acetylcysteine (NAC)/g bodyweight from 0.5 dpc to 14.5 dpc, in the water, and IP injection of bC^H^; bC^L^, low dose, 175 μg/day by IP injection from 6.5–9.5 dpc; apoAL, β-apo-10′-carotenal; apoAL^4dh^, 800 μg/day by IP injection from 6.5–9.5 dpc; apoAL^3dh^, 800 μg/day from 7.5–9.5 dpc either in the food or by IP injection; apoAL^3dl^, 400 μg/day from 7.5–9.5 dpc either in the food or by IP injection.

### BCO2 prevents infertility induced under severe vitamin A deficiency by high doses of β-carotene

Considering the mitochondrial localization of BCO2 and its prevention of oxidative stress and apoptosis in adult tissues by scavenging excess carotenoids^15,16^, we investigated whether BCO2 performs a similar role in embryos. We supplemented pregnant *Bco2*^−/−^*Rbp4*^−/−^ females, on a VAD diet during pregnancy, with two different doses of β-carotene (High: 800 μg/day by intraperitoneal [IP] injection from 6.5–9.5 dpc^11^, or Low: 175 μg/day by IP injection from 6.5–9.5 dpc). Despite the maternal and embryonic expression of BCO1, which could have converted β-carotene into retinoids to support embryogenesis^11^, maternal supplementation with β-carotene compromised the fertility of the *Bco2*^−/−^*Rbp4*^−/−^ females in a dose-dependent manner, resulting in a high rate of unsuccessful pregnancies (87%) at the higher dose (Fig. 1B). Note that fertility is defined here as the ability of a female mouse presenting a vaginal plug - a sign of successful mating - to carry at least one embryo at 14.5 dpc. Interestingly, when the vitamin A-deprived *Bco2*^−/−^*Rbp4*^−/−^ dams supplemented with the high dose of β-carotene were also supplemented with the antioxidant N-acetylcysteine (NAC) throughout gestation, the infertility was completely prevented (Fig. 1B; 0% unsuccessful pregnancies).

Of note, vitamin A deficiency itself is associated with oxidative stress^21–23^. When the dams were fed a VAD diet, the mRNA levels of several oxidative stress response genes were slightly but significantly higher in *Bco2*^−/−^*Rbp4*^−/−^ than in *Rbp4*^−/−^ embryos at 14.5 dpc (Fig. 1C), suggesting that the double-knockout embryos experienced higher levels of oxidative stress than their *Rbp4*^−/−^ counterparts by virtue of their more severe vitamin A deficiency (Fig. 1A). β-carotene treatment of *Bco2*^−/−^*Rbp4*^−/−^ mice from 6.5–9.5 dpc, however, did not exacerbate these mRNA expression changes at 14.5 dpc (Fig. 1C). To determine whether the toxicity of β-carotene in BCO2-deficient mice was indeed related to vitamin A deficiency, we examined the effects of β-carotene supplementation in *Bco2*^−/−^ (single-knockout) mice on a VAD diet. Dietary vitamin A deprivation of *Bco2*^−/−^ dams during gestation did not result in vitamin A deficiency (as expected, given the expression of RBP4^12^; data not shown). However, unsuccessful pregnancies were still observed in the *Bco2*^−/−^ strain upon maternal β-carotene supplementation, although at a lower frequency (64%) than in the *Bco2*^−/−^*Rbp4*^−/−^ strain (87%, see Fig. 1B).

Overall, these data confirm that the toxicity of β-carotene is greater when accompanied by vitamin-A-deficiency-induced oxidative stress. When β-carotene is provided during severe vitamin A deficiency, BCO2 performs a specific non-redundant function that cannot be overcome by the generation of retinoids *via* BCO1: it prevents infertility, likely by cleaving β-carotene (and/or its derivatives) to counteract its pro-oxidant effects at high doses^24^.

### β-apo-10′-carotenal promotes normal development during severe gestational vitamin A deficiency

**Figure 3:**
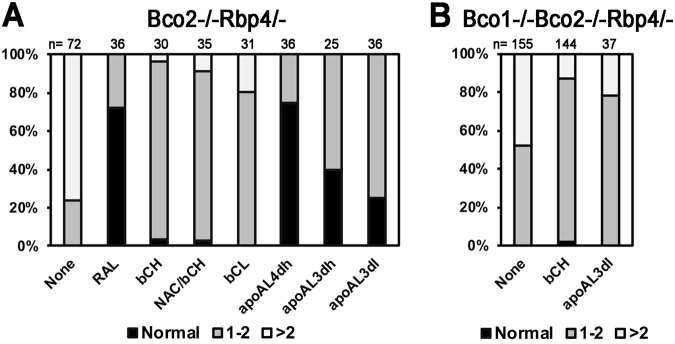
Percent of different gross morphological defects in 14.5-dpc embryos from various strains of dams fed the vitamin A-deficient diet during pregnancy and under various regimens of maternal supplementation. Embryonic genotype as maternal genotype. The X-axis displays the regimen of maternal supplementation: None, no supplementation; RAL, retinaldehyde, 1600 μg/day in diet from 7.5–9.5 dpc; β-carotene (bC); bC^H^, high dose, 800 μg/day by IP injection from 6.5–9.5 dpc; NAC/bC^H^, 0.5 mg of N-acetylcysteine (NAC)/g bodyweight from 0.5 dpc to 14.5 dpc, in the water, and IP injection of bC^H^; bC^L^, low dose, 175 μg/day by IP injection from 6.5–9.5 dpc; apoAL, β-apo-10′-carotenal; apoAL^4dh^, 800 μg/day by IP injection from 6.5–9.5 dpc; apoAL^3dh^, 800 μg/day from 7.5–9.5 dpc either in the food or by IP injection; apoAL^3dl^, 400 μg/day from 7.5–9.5 dpc either in the food or by IP injection. For each treatment, the percentages of embryos with no defects (normal), 1–2 defects, and >2 defects are shown. Defects included abnormal eyes, peripheral edemas, cleft face/palate, exencephaly, and spinal defects, as shown and described in Fig. 2. (**A**) *Bco2*^−/−^*Rbp4*^−/−^ embryos. n, as in Table 2. (**B**) *Bco1*^−/−^*Bco2*^−/−^*Rbp4*^−/−^ embryos. n, as in Table 3.

We next evaluated the phenotypes of the limited number of embryos recovered at mid-gestation from the vitamin A-deprived *Bco2*^−/−^*Rbp4*^−/−^ dams supplemented with the high-dose regimen of β-carotene. Intriguingly, despite the maternal and embryonic expression of BCO1, these embryos were still malformed following maternal β-carotene supplementation. The gross morphological abnormalities included abnormal eyes, peripheral edemas, cleft face/palate, exencephaly and skeletal defects (Fig. 2B–F), and in most cases, multiple defects were present in any given embryo. Figure 3A displays the percent of embryos with 1–2 gross malformations vs. the percent of those with >2 morphological defects. Compared to the unsupplemented condition, maternal β-carotene administration resulted in a lower percent of *Bco2*^−/−^*Rbp4*^−/−^ embryos with >2 defects (and thus a larger percent with 1–2 defects). However, four embryos displayed skeletal malformations such as twisted spines and shortened hind limbs (Fig. 2F) that were not observed in the unsupplemented animals or in mice administered retinaldehyde. Only 3% (a single embryo) displayed a normal gross morphology (Table 2c and Fig. 3A).

Furthermore, maternal co-supplementation with the antioxidant NAC throughout gestation and the high dose of β-carotene from 6.5–9.5 dpc (as described above; Table 2d and Fig. 3A) or with the lower dose of β-carotene from 6.5–9.5 dpc (Table 2e and Fig. 3A) did not result in normal embryos at mid-gestation (only one embryo in the case of NAC supplementation; Table 2d and Fig. 3A). These data support our interpretation that the β-carotene cleavage activity of not only BCO1^11^, but also BCO2, is required to facilitate embryonic development during vitamin A deficiency when β-carotene is supplemented. Of note, *Bco2*^−/−^ (single knockout) embryos from dams fed the VAD diet and supplemented with β-carotene from 6.5–9.5 dpc developed normally (51 normal embryos from 8 dams), indicating that this role of BCO2 may be specific to conditions of vitamin A deficiency (in this case, modeled on the *Rbp4*^−/−^ background).

**Figure 4:**
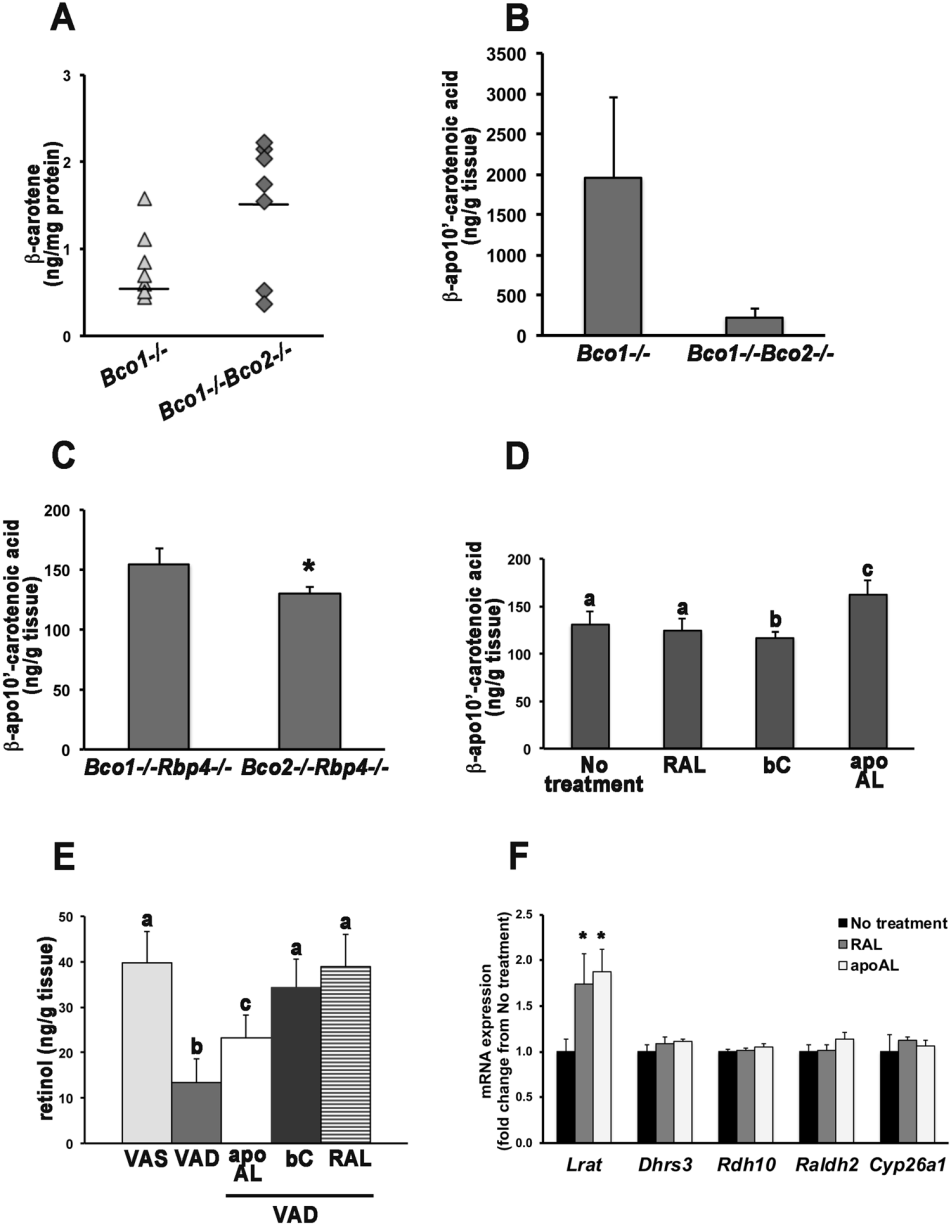
BCO2 generates β-apo-10′-carotenal, which promotes normal embryonic development during severe gestational vitamin A deficiency. (**A**) HPLC analysis of β-carotene in hepatic mitochondria from *Bco1*^−/−^ and *Bco1*^−/−^*Bco2*^−/−^ females fed a regular chow diet and sacrificed 5 hours after IP injection with 800 μg β-carotene. n = 7/group. (**B**) LC-MS analysis of β-apo-10′-carotenoic acid in *Bco1*^−/−^ and *Bco1*^−/−^*Bco2*^−/−^ 14.5-dpc embryos from dams maintained on a regular chow diet and injected with β-carotene (800 μg, IP) at 13.5 dpc. n = 3/group. Data are means ± SEMs. (**C**) LC-MS analysis of β-apo-10′-carotenoic acid in *Bco1*^−/−^*Rbp4*^−/−^ and *Bco2*^−/−^*Rbp4*^−/−^ 14.5-dpc embryos from dams fed a VAD diet from 0.5 dpc. n = 4/group. Data are means ± SDs. *p < 0.05. (**D**) LC-MS analysis of β-apo-10′-carotenoic acid in *Bco2*^−/−^*Rbp4*^−/−^ embryos from dams fed a VAD diet from 0.5 dpc, unsupplemented (no treatment) or supplemented with β-apo-10′-carotenal (apoAL; 400 μg/day from 7.5–9.5 dpc by IP injection or diet), β-carotene (bC; 800 μg/day from 6.5–9.5 dpc by IP injection) or retinaldehyde (RAL; 1600 μg/day from 7.5–9.5 dpc in the diet). n = 4/group. Data are means ± SDs. Labeled means without a common letter differ, p < 0.05. (**E**) HPLC analysis of retinol in *Bco2*^−/−^*Rbp4*^−/−^ embryos from dams on a VAS, VAD or VAD diet supplemented with apoAL, bC or RAL. Supplementation regimens as in (**D**). n = 3–9/group. Data are means ± SDs. Labeled means without a common letter differ, p < 0.05. (**F**) qPCR analysis of key genes that regulate retinoid homeostasis in 14.5-dpc embryos from *Bco2*^−/−^*Rbp4*^−/−^ dams fed the VAD diet from 0.5 dpc, with or without β-apo-10′-carotenal (apoAL; 800 μg/day from 6.5–9.5 dpc by IP injection) or retinaldehyde (1600 μg/day from 7.5–9.5 dpc in the diet) supplementation. *Bco2*^−/−^*Rbp4*^−/−^ embryos from dams only on the VAD diet from 0.5 dpc were set as the calibrator at 1. Data are means ± SDs fold of *Bco2*^−/−^*Rbp4*^−/−^; n = 4–5 embryos/group (from 3–4 dams/group). Statistical analysis performed by one-way ANOVA. *p < 0.05 vs. the calibrator group.

We therefore asked whether, in addition to the scavenging action of BCO2, its apocarotenoid-generating action *per se* was required. First, we confirmed that BCO2 controls the mitochondrial carotenoid concentration^15,16,25^. Indeed, hepatic mitochondrial levels of β-carotene tended to be higher in *Bco1*^−/−^*Bco2*^−/−^ than in *Bco1*^−/−^ mice supplemented once with β-carotene (Fig. 4A). Next, we tested whether embryonic apocarotenoid levels were affected by BCO2 expression. We performed liquid chromatography-mass spectrometry (LC-MS) analysis of 14.5-dpc *Bco1*^−/−^ and *Bco1*^−/−^*Bco2*^−/−^ embryos from dams maintained on a VAS diet (regular chow) and injected with β-carotene at 13.5 dpc. While we did not detect β-apo-10′-carotenol (reported by^10^) or β-apo-10′-carotenal (the expected primary product^13^), we detected β-apo-10′-carotenoic acid, the levels of which tended to be lower in β-carotene-treated *Bco1*^−/−^*Bco2*^−/−^ embryos than in *Bco1*^−/−^ embryos (Fig. 4B). These data confirmed that the ability of the embryos to generate β-apo-10′-carotenoids from β-carotene depends on BCO2, as in adult tissues^10^.

We also detected β-apo-10′-carotenoic acid in *Bco2*^−/−^*Rbp4*^−/−^ embryos from unsupplemented dams fed the VAD diet during pregnancy (Fig. 4C), indicating that β-apocarotenoids absorbed from carotenoid-containing chow diets^26^ (such as the diet fed to the dams prior to the VAD diet during gestation) can cross the placental barrier. The embryonic concentration of β-apo-10′-carotenoic acid was significantly lower in *Bco2*^−/−^*Rbp4*^−/−^ embryos than in *Bco1*^−/−^*Rbp4*^−/−^ embryos^11^ from dams fed the VAD diet during pregnancy (Fig. 4C), indicating that the baseline tissue concentration of this dietary-derived β-apocarotenoid^26^ depends on which carotenoid cleavage enzyme is expressed. Interestingly, the *Bco2*^−/−^*Rbp4*^−/−^ embryos exhibited a more severe phenotype than the *Bco1*^−/−^*Rbp4*^−/−^ embryos^11^ (in this study, abnormal eye, edema and cleft face/palate: 76% in the *Bco2*^−/−^*Rbp4*^−/−^ vs. 22% in the *Bco1*^−/−^*Rbp4*^−/−^). Given the notion that β-apocarotenals can be oxidized to the corresponding β-apocarotenoic acids in the mitochondrial and microsomal fractions of rat liver homogenates^17^, we speculated that the embryonic phenotype of the *Bco2*^−/−^*Rbp4*^−/−^ was due to low levels of β-apo-10′-carotenal, together with the limited availability of retinoids.

**Figure 5:**
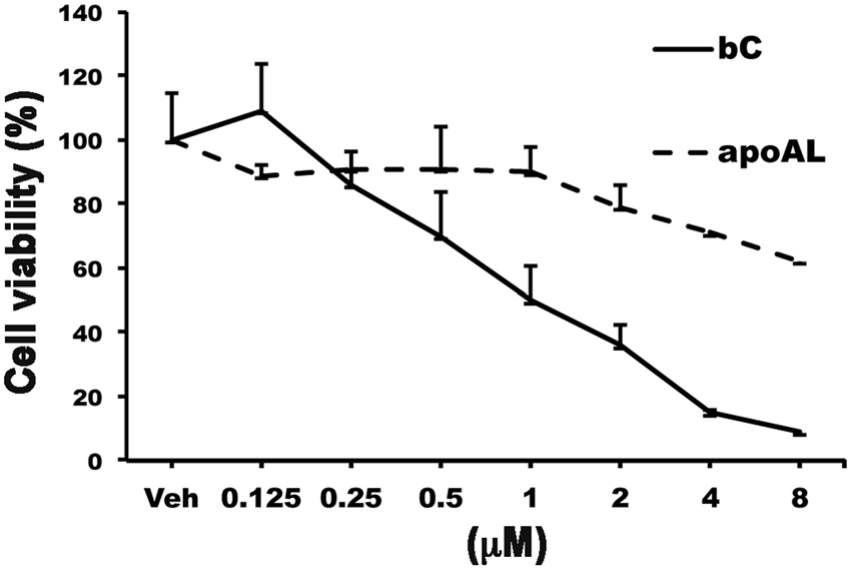
Cellular viability in WT mouse embryonic fibroblasts treated with β-carotene or β-apo-10′-carotenal. WT mouse embryonic fibroblasts in serum-free medium were treated overnight with β-carotene (bC), β-apo-10′-carotenal (apoAL), or Vehicle (Veh). The next day, the cells were incubated with WST-1, and the absorbance was read at 450 nm and 630 nm. Data are presented as means ± SDs from triplicate samples.

To test this hypothesis, we administered to the *Bco2*^−/−^*Rbp4*^−/−^ dams on a VAD diet three different regimens of β-apo-10′-carotenal by IP injection or dietary supplementation: four-day high (800 μg/day from 6.5–9.5 dpc), three-day high (800 μg/day from 7.5–9.5 dpc) and three-day low (400 μg/day from 7.5–9.5 dpc). The pregnancy success rate was 100% in dams treated with β-apo-10′-carotenal, regardless of the regimen (Fig. 1B), suggesting that β-apo-10′-carotenal is non-toxic, unlike β-carotene^15,16,24^. Indeed, viability assays of WT mouse embryonic fibroblasts in serum-free medium (i.e., also deprived of vitamin A), treated for 24 hours with different doses of β-carotene or β-apo-10′-carotenal, revealed that cells treated with β-apo-10′-carotenal were less susceptible to cell death than those treated with β-carotene (Fig. 5).

In addition to restoring fertility (Fig. 1B), the aforementioned maternal β-apo-10′-carotenal supplementation regimens dose-dependently improved the phenotype of the *Bco2*^−/−^*Rbp4*^−/−^ embryos. The percentage of phenotypically normal embryos reached 77% with the four-day high regimen (Table 2f–h and Fig. 2H), and the remaining embryos only displayed 1–2 morphological defects (Fig. 3A), representing a substantial improvement of the phenotype compared to embryos from unsupplemented dams or from dams administered β-carotene (Fig. 3A). Further, the levels of β-apo-10′-carotenoic acid in *Bco2*^−/−^*Rbp4*^−/−^ embryos at 14.5 dpc increased upon maternal β-apo-10′-carotenal supplementation (Fig. 4D). Remarkably, β-apo-10′-carotenal supplementation of *Bco2*^−/−^*Rbp4*^−/−^ dams on the VAD diet during gestation (four-day high) resulted in the birth of phenotypically normal live pups, which survived to adulthood (32 out of 55 live births) and were fertile (14 of the rescued pups successfully mated at three months of age).

All together, these results strongly indicate that β-apo-10′-carotenal can promote normal development during severe gestational vitamin A deficiency.

### β-apo-10′-carotenal as a retinoid precursor

As β-apo-10′-carotenal-supplemented *Bco2*^−/−^*Rbp4*^−/−^ dams expressed BCO1, their embryos could have been rescued through the BCO1-catalyzed conversion of β-apo-10′-carotenal to retinaldehyde^10,14^, followed by oxidation to retinoic acid or reduction to retinol. Indeed, supplementation of *Bco2*^−/−^*Rbp4*^−/−^ dams with β-apo-10′-carotenal increased embryonic retinol levels (Fig. 4E). Moreover, the mRNA levels of key regulators of retinoid homeostasis in 14.5-dpc *Bco2*^−/−^*Rbp4*^−/−^ embryos from dams on the VAD diet, with or without β-apo-10′-carotenal supplementation, indicated that maternal β-apo-10′-carotenal supplementation significantly increased the expression of embryonic lecithin:retinol acyltransferase (*Lrat*), the gene coding for the enzyme that synthesizes retinyl esters, the storage form of vitamin A^27^ (Fig. 4F). Importantly, similar upregulation of *Lrat* was observed in *Bco2*^−/−^*Rbp4*^−/−^ embryos from dams supplemented directly with retinaldehyde (Fig. 4F). These results suggest that β-apo-10′-carotenal is indeed converted into retinoids in the embryo, and that retinoid store formation may be induced to prevent the synthesis of excess retinoic acid from retinaldehyde.

We obtained further evidence of this β-apo-10′-carotenoid-mediated retinoid salvage pathway by using mice lacking both carotenoid cleavage enzymes on the *Rbp4*^−/−^ background (*Bco1*^−/−^*Bco2*^−/−^*Rbp4*^−/−^). When these mice were fed the purified VAD diet during gestation, their embryos also exhibited developmental defects (Table 3a). Maternal β-carotene supplementation of the triple-knockout dams deprived of dietary vitamin A increased the percentage of unsuccessful pregnancies (Fig. 1B) and did not result in normal embryos (only one out of 144 was grossly morphologically normal) (Table 3b), although it reduced the severity of the congenital malformations (Fig. 3B). Furthermore, supplementation of *Bco1*^−/−^*Bco2*^−/−^*Rbp4*^−/−^ dams with β-apo-10′-carotenal improved the pregnancy rate (Fig. 1B), but still did not yield normal embryos (Table 3c), although it also reduced the severity of the congenital defects (Fig. 3B). These data support the possibility that some of the β-apo-10′-carotenal may have been converted to retinoids *via* BCO1 in the supplemented *Bco2*^−/−^*Rbp4*^−/−^ mice.

**Table 3:** Phenotype distribution of embryos at 14.5 dpc from *Bco1*^−/−^*Bco2*^−/−^*Rbp4*^−/−^ dams fed the vitamin A-deficient diet during pregnancy and under various regimens of maternal supplementation.

Supplem.	n [embryos (dams)]	Normal	Malformed
a. None	155 (22)	0%	100%
b. bC^H^	144 (15)	2%	98%
c. apoAL^3dl^	37 (6)	0%	100%

Embryonic genotype is as the maternal genotype. Supplem., regimen of maternal supplementation: None, no supplementation; bC^H^, β-carotene high dose (800 μg/day by IP injection from 6.5–9.5 dpc); apoAL^3dl^, β-apo-10′-carotenal (400 μg/day) from 7.5–9.5 dpc either in the food or by IP injection.

## Discussion

For more than a decade, there has been great interest in the two carotenoid cleavage enzymes in vertebrates and their relative contribution to animal metabolism. As both enzymes are expressed in most tissues^28–30^, it was logical to assume that their functions and substrate preferences would not entirely overlap. Over time, BCO1 emerged as the dominant retinoid-generating enzyme in both adult^9^ and embryonic tissues^11^. This enzyme symmetrically cleaves (at their 15,15′ bonds) carotenoids with at least one unsubstituted β-ionone ring^28^. In contrast, BCO2 was found to asymmetrically cleave a broad array of substrates at their 9′,10′ or 9,10 bonds, allowing for substituted rings in compounds including carotenes^13,15^, xanthophylls, and even certain retinoids^16^. The contribution of BCO2 to retinoid homeostasis has been considered minimal, given its allowance for a variety of substrates and the lack of retinoid deficiency in *Bco2*^−/−^ mice^15^. Only recently has BCO2 been identified as a potentially critical enzyme in retinoid metabolism, due to its ability to cleave β-carotene and generate β-apo-10′-carotenal^13^, which subsequently can be converted to retinaldehyde by BCO1^10^.

Interestingly, our experiments demonstrated that BCO2 deficiency exacerbated embryonic vitamin A deficiency. Specifically, the lack of BCO2 on the vitamin A deficiency-susceptible *Rbp4*^−/−^ background reduced embryonic retinol levels, regardless of the vitamin A content of the diet (Fig. 1A). Accordingly, the phenotype of the *Bco2*^−/−^*Rbp4*^−/−^ embryos from dams on the VAD diet during pregnancy was more severe than that of *Rbp4*^−/−^ embryos from dams on a similar dietary regimen, as in addition to eye defects and peripheral edemas (the hallmarks of embryonic vitamin A deficiency in the *Rbp4*^−/−^ mice^12^), they displayed other congenital malformations (Figs 2 and 3A). This is not the first evidence that a carotenoid cleavage enzyme can impact retinoid homeostasis. Indeed, we previously reported that *Bco1*^−/−^ embryos had lower retinyl ester levels than WT embryos, accompanied by lower mRNA levels and enzymatic activity of LRAT, the main enzyme that synthesizes retinyl esters for storage^11^. BCO1 deficiency also attenuated the embryonic concentrations of triglycerides and cholesteryl esters, and the mRNA levels of the corresponding acyltransferases (*Acat1*, *Dgat2*, and *Lcat*)^31^. We found that retinyl ester levels were reduced in embryos lacking *Bco2* (Supplementary Table S1), but the mRNA levels of *Acat1*, *Dgat2* and *Lcat* did not differ from those of WT embryos (Supplementary Fig. S2). Therefore, it appears that BCO1 and BCO2 affect embryonic retinoid homeostasis differently, by mechanisms that remain to be determined. Whether the contribution of BCO2 to embryonic retinoid homeostasis is independent of its ability to cleave carotenoid substrate(s) cannot be unequivocally established at this time. Although our purified *experimental* mouse diets were carotenoid-free, it is unclear to what extent the small amounts of β-carotene and β-apocarotenoids in the regular chow *maintenance* diet^26^ prior to the provision of the purified experimental diets may have affected the phenotype of the mice lacking BCO2.

In adult mammalian tissues, BCO2 has been proposed to protect mitochondria from oxidative stress by scavenging excess carotenoids (including β-carotene) and their derivatives (dehydrocarotenoids) generated *via* spontaneous oxidation^15,16^. It is striking that high-dose β-carotene supplementation resulted in an 87% unsuccessful pregnancy rate in *Bco2*^−/−^*Rbp4*^−/−^ mice on the VAD diet, while this infertility was completely reverted in the presence of the antioxidant NAC (Fig. 1B). These results suggest that adequate antioxidative defenses, including BCO2-dependent scavenging of toxic carotenoids, are important guarantors of successful embryogenesis. Notably, vitamin A deficiency itself enhances oxidative stress, as indicated by the changes in the activity of catalase, glutathione-S-transferase, glutathione peroxidase, DT diaphorase and urate oxidase in the livers of vitamin A-deficient rats and mice^21–23^. We found that several oxidative stress response genes were slightly but significantly upregulated in *Bco2*^−/−^*Rbp4*^−/−^ compared with *Rbp4*^−/−^ embryos on the VAD diet (Fig. 1C). Whereas *Sod1* and *Gpx1* encode antioxidant enzymes^32^, their upregulation, accompanied by the increased expression of genes encoding pro-oxidant enzymes (*Duox1* and *Alcat1*^33^), may reflect an attempt of the *Bco2*^−/−^*Rbp4*^−/−^ embryos to offset a higher level of oxidative stress^34^ than their *Rbp4*^−/−^ counterparts, due to the more severe vitamin A-deficient status of the *Bco2*^−/−^*Rbp4*^−/−^. Although the 14.5-dpc embryonic mRNA levels of oxidative stress response genes were not further modulated in *Bco2*^−/−^*Rbp4*^−/−^ mice following β-carotene supplementation, it is still possible that oxidative stress was higher during the treatment window (5–8 days earlier). Future studies will better define the oxidative stress status of these embryos at an earlier stage of gestation (i.e., 9.5 dpc).

In female *Bco2*^−/−^*Rbp4*^−/−^ mice with detectable vaginal plugs (an indication of successful mating) and supplemented with β-carotene, signs of implantations were visible in the uterus at dissection (14.5 dpc); thus, it seems that the β-carotene toxicity occurred relatively early during gestation. Under our experimental conditions, β-carotene was efficiently delivered to the uterus, where BCO2 is expressed^28^ (22.2 ± 3.7 μg β-carotene/g; n = 3 *Bco2*^−/−^*Rbp4*^−/−^ virgin females on regular chow diet, supplemented with the high dose of β-carotene for four days and sacrificed 4 hours after the last injection). However, the total nuclear DNA contents of 8-oxo-7,8-dihydroguanine (8-oxoG), the most commonly formed oxidative DNA lesion in cells and a marker of oxidative stress^35^, were similar in the uteri of β-carotene-supplemented and vehicle-treated *Bco2*^−/−^*Rbp4*^−/−^ females (59.8 ± 2.9 vs. 63.5 ± 4.9 fmol/μg DNA, respectively), suggesting that the contribution of maternal (uterine) BCO2 deficiency to the oxidative stress-induced infertility^15,16^ was negligible, at least under these conditions. Further investigations should unequivocally establish the maternal vs. embryonic contribution to the infertility in this mouse model in relation to vitamin A deficiency and oxidative stress.

The infertility of the vitamin A-deficient *Bco2*^−/−^*Rbp4*^−/−^ dams supplemented with β-carotene was clearly dose-dependent (Fig. 1B), as was the toxicity of β-carotene in a mouse embryonic fibroblast viability assay (Fig. 5). Further, we have observed an increased incidence of unsuccessful pregnancies upon β-carotene treatment of other models of embryonic vitamin A deficiency with intact carotenoid cleavage enzymes, including mice lacking both RBP4 and LRAT (*Lrat*^−/−^*Rbp4*^−/−^^36^; 50% unsuccessful pregnancies). Thus, it appears that β-carotene toxicity can occur when BCO2 is inactive (i.e., in *Bco2*^−/−^ strains) or when the enzyme is saturated at high doses of substrate (i.e., in strains that do express BCO2).

The exact mechanisms whereby excessive carotenoids induce toxicity have not been fully elucidated, although a potential role has been ascribed to carotenoid derivatives generated upon spontaneous carotenoid oxidation, at least in adult tissues. In the first report on *Bco2*^−/−^ mice^15^, the authors demonstrated that BCO2 deficiency coupled with dietary carotenoid (xanthophyll) supplementation led to the formation of 3-dehydrocarotenoids, their accumulation in mitochondria, a reduction in hepatic mitochondrial ADP-dependent (state 3) respiration compared to untreated *Bco2*^−/−^, and an upregulation of hepatic SOD2 (MnSOD), HIF1-α, phospho-AKT and phospho-MAPK protein expression compared to WT mice. In HepG2 cells (which do not express BCO2) treated with β-carotene or the same dehydrocarotenoids detected in xanthophyll-fed *Bco2*^−/−^ mice, ROS production was induced and the mitochondrial membrane potential decreased, while these outcomes were ameliorated when the cells were transfected with a plasmid expressing *Bco2*. These results suggested that the accumulation of (dehydro)carotenoids in mitochondria could directly affect the electron transport chain, thus triggering oxidative stress^15^. In a later study^16^, the same group demonstrated that the oxidative stress in carotenoid-treated HepG2 cells led to cytochrome c release, pro-caspase 3 and PARP1 cleavage, and chromatin condensation, all hallmarks of the apoptotic pathway.

In *Bco2*^−/−^*Rbp4*^−/−^ embryos, the toxicity of β-carotene appeared to outweigh its beneficial effects, since the ability to generate retinoids (Fig. 4E) from β-carotene *via* BCO1 was not sufficient to ensure the survival or normal development of these embryos upon maternal vitamin A deprivation (Fig. 1B and Table 2c,e). From this perspective, it appears that the ability to scavenge β-carotene or its spontaneous oxidative cleavage products by BCO2 is as important for embryonic development as the ability to cleave β-carotene by BCO1, at least when β-carotene is the only available vitamin A source. During vitamin A deficiency, when oxidative stress is already high, the relative importance of BCO2 during embryogenesis may be greater than it is under more normal circumstances. Evidently, by supplementing *Bco2*^−/−^*Rbp4*^−/−^ dams with β-apo-10′-carotenal (the product of β-carotene cleavage by BCO2), we were able to bypass the mitochondrial toxicity of β-carotene and provide a direct substrate for BCO1 to generate retinoids^10^. We demonstrated for the first time that β-apo-10′-carotenal can dose-dependently reverse embryonic vitamin A deficiency syndrome in a mouse model, and is minimally toxic to cells (Fig. 5). At the very least, this is further evidence of the coordinated action of BCO2 and BCO1^10^.

Interestingly, even a lower dose of β-carotene or co-treatment with an antioxidant did not result in more than one normal *Bco2*^−/−^*Rbp4*^−/−^ embryo at mid-gestation, despite the fact that the infertility was rescued. This may indicate that β-carotene toxicity was not the only contributor to the *Bco2*^−/−^*Rbp4*^−/−^ embryonic phenotype, and perhaps that retinoids were not the only deficient metabolites in these mice (i.e., that the inability to generate β-apo-10′-carotenal itself was detrimental). Furthermore, even in the absence of both cleavage enzymes (in the *Bco1*^−/−^*Bco2*^−/−^*Rbp4*^−/−^), supplementation with β-apo-10′-carotenal reduced the severity of the congenital defects, although it did not yield normal embryos (Table 3c and Fig. 3B). This raises the question of whether β-apo-10′-carotenal also contributes to embryonic development *per se*, by BCO1-independent mechanisms. This possibility warrants investigation.

On the other hand, it should be noted that even β-carotene supplementation partially improved the phenotype of vitamin A-deficient embryos lacking both carotenoid cleavage enzymes (Table 3b and Fig. 3B). It is generally assumed that a third mammalian carotenoid oxygenase does not exist, as BCO1 and BCO2 share amino acid sequence homology only with each other and a retinoid isomerase expressed only in the eye (RPE65)^37^. Therefore, the well-established phenomenon of spontaneous carotenoid oxidation is the most likely reason for the phenotype improvement of the supplemented *Bco1*^−/−^*Bco2*^−/−^*Rbp4*^−/−^. Indeed, high-performance liquid chromatography (HPLC) analysis of our β-carotene solutions revealed a variety of compounds indicative of β-carotene oxidation, which were detected at even higher levels in the sera and livers of injected mice (Supplementary Fig. S3). The conversion of small amounts of these spontaneously generated β-carotene oxidation products to retinoic acid (by chain shortening^38^, given the lack of BCO1) could have facilitated vitamin A-dependent developmental processes and thus partially improved the embryonic phenotype. Whatever spontaneous oxidation mechanisms are at work, they appear to be highly inefficient, as they resulted in a miniscule number of normal embryos (Table 3b and Fig. 3B).

In conclusion, our data suggest that β-apo-10′-carotenal, the product of BCO2-mediated β-carotene cleavage, is a potent retinoid precursor that strongly supports survival and development. Indeed, in *Bco2*^−/−^*Rbp4*^−/−^ dams on the VAD diet during pregnancy, β-apo-10′-carotenal supplementation for only four days during early-to-mid gestation resulted in normal live pups which survived to adulthood and were fertile. These results should be considered in light of the astonishing number of children and pregnant women who remain vitamin A-deficient worldwide, despite the well-meaning efforts of intervention programs employing vitamin A and/or β-carotene^5,6,39^. In a recent review of major programs of vitamin A or β-carotene supplementation during pregnancy or lactation in areas with endemic vitamin A deficiency, it was concluded that the findings did not support a role for antenatal vitamin A supplementation to reduce maternal or perinatal mortality for various reasons, including lack of proper assessment of baseline vitamin A status^40^. However, in the studies employing β-carotene, the provitamin A carotenoid was used at a relatively high dose (42 mg) (reviewed in^40^). Our data suggest that β-carotene supplementation could be detrimental for embryos developing from severely vitamin A-deficient mothers, leading to early embryonic lethality (i.e., miscarriage or infertility). Unfortunately, human data linking vitamin A deficiency to infertility or increased risk of miscarriage are scarce, mainly because these outcomes are extremely difficult to track in countries were the health system is inefficient or non-existent. A meta-analysis indicated that supplementation of vitamin A-deficient women with retinoids and/or β-carotene did not affect the rates of miscarriage and fetal loss^41^. However, in these studies there was a wide range of enrollment stages, mostly outside the first trimester, and in some of them women were supplemented with retinoids rather than β-carotene, further highlighting the scarcity of studies on early embryonic lethality. We have also demonstrated that β-apo-10′-carotenal is more effective than β-carotene in promoting normal development in a mouse model of embryonic vitamin A deficiency. Future studies should evaluate the efficacy of β-apo-10′-carotenal in other experimental models of vitamin A deficiency and subsequently in clinical trials, to consider the possible inclusion of β-apo-10′-carotenal in nutritional interventions for pregnant women to sustain normal embryonic development under vitamin A deficiency.

## Methods

### Animals

Mice of the following genotypes were used in this study: wild-type (WT), *Rbp4*^−/−^ ^42^, *Bco1*^−/−^*Rbp4*^−/−^ ^11^, *Bco1*^−/−^ ^9,43^, *Bco2*^−/−^ ^15^, *Bco1*^−/−^*Bco2*^−/−^, *Bco2*^−/−^*Rbp4*^−/−^, and *Bco1*^−/−^*Bco2*^−/−^*Rbp4*^−/−^. The three latter strains were obtained by crossing *Bco1*^−/−^*Rbp4*^−/−^ with *Bco2*^−/−^ mice. The resulting *Bco1*^+/−^*Bco2*^+/−^*Rbp4*^+/−^ mice of the F1 and subsequent generations were crossed, and mice of the three above-mentioned genotypes were obtained at the expected Mendelian ratios. Genotypes were confirmed as reported^9,11,15,42,43^. All mice harbored a mixed genetic background (C57BL/6 × Sv/129). Due to the high dietary vitamin A requirements of the *Rbp4*^−/−^ ^44^, all mice were maintained throughout life on a regular chow vitamin A-sufficient diet containing 18 IU vitamin A/g (Prolab Isopro RMH3000 5p75) until the time of nutritional manipulation. This diet also contained a small amount of carotenoids (<1.2 μg/g). Mice had access *ad libitum* to diet and water, and were maintained on a 12-hour dark-light cycle between 7:00 PM and 7:00 AM. All dams were sacrificed by CO_2_ inhalation at 14.5 dpc between 9:00 and 11:30 AM. At sacrifice, maternal serum, liver and embryos were collected, immediately frozen on dry ice, and stored at −80 °C until further analysis. All experiments were conducted in accordance with the National Institutes of Health Guide for the Care and Use of Laboratory Animals and were reviewed and approved by the Rutgers University Animal Care and Use Committee. At the time of dissection, the gross morphology of the embryos was documented. Embryos were classified as normal if they phenotypically resembled WT embryos from dams maintained on a VAS diet. Abnormal embryos exhibited one or more of the following features: small or absent eye, peripheral edema, abnormal mid-facial region (snout foreshortened and divided by a sagittal median cleft, prolabium absent, maxillary process bearing whiskers separated by a larger-than-normal distance), exencephaly (exposed brain), and twisted spine.

### Nutritional manipulation and supplementation protocols

At approximately 90 days of age, females were mated with males of their respective genotypes, and the morning of vaginal plug detection was set as the beginning of pregnancy (0.5 dpc). From 0.5 dpc until 14.5 dpc, dams were fed a purified vitamin A-deficient and carotenoid-free diet (Research Diets, <0.2 IU/g vitamin A, 0 μg/g carotenoids), and dams were assigned to various treatment groups.

#### Retinaldehyde supplementation

A group of *Bco2*^−/−^*Rbp4*^−/−^ dams was maintained on the vitamin A-deficient diet throughout gestation, but supplemented with retinaldehyde (1600 μg/day) from 7.5–9.5 dpc, as published^20^. Briefly, a 50 mg/mL ethanolic solution of retinaldehyde was diluted in a mixture of pulverized vitamin A-deficient, carotenoid-free diet and water (1:1, g: mL) to yield the final dose of 400 μg/g diet, or 1600 μg/day, assuming a daily intake of 4 g food.

#### β-carotene supplementation

As the highly efficient intestinal carotenoid cleavage activity in mice prevents the absorption of intact β-carotene^45^, we bypassed the intestinal barrier by injecting β-carotene IP daily from 6.5–9.5 dpc (the period of most organogenesis in mice^46^). The β-carotene solution was prepared as described previously^44^. Briefly, β-carotene (Type I, Sigma Aldrich) was dissolved in vehicle by sequential addition of ethanol, Kolliphor (C5135, Sigma Aldrich) and phosphate-buffered saline (PBS) at a ratio of 1:11:18, and the concentration was determined spectrophotometrically at 450 nm. In each daily injection (6.5–9.5 dpc), an average dose of 800 μg (high dose) or 175 μg (low dose) of β-carotene was administered (~36 μg β-carotene/g bodyweight, assuming ~22 g bodyweight, for the high dose).

#### N-acetylcysteine (NAC) supplementation

Additional pregnant *Bco2*^−/−^*Rbp4*^−/−^ dams were provided with the antioxidant NAC at a dose of 0.5 mg/g bodyweight in their drinking water from 0.5–14.5 dpc (modified from^47^). Water intake was monitored throughout the experiment, and water was replaced every two to three days. These females were fed the vitamin A-deficient diet from 0.5–14.5 dpc and injected with β-carotene IP from 6.5–9.5 dpc.

#### β-apo-10′-carotenal supplementation

Groups of dams also were fed the vitamin A-deficient diet throughout gestation, and supplemented with β-apo-10′-carotenal by IP injection according to one of the following regimens: four-day high (4 dh; 800 μg/day from 6.5–9.5 dpc), three-day high (3 dh; 800 μg/day from 7.5–9.5 dpc) and three-day low (3 dl; 400 μg/day from 7.5–9.5 dpc). The IP solution was prepared and injected as described above for β-carotene. Certain dams received the supplement in their food (400 μg/day [n = 1] or 800 μg/day [n = 3] from 7.5–9.5 dpc). Briefly, an ethanolic solution of β-apo-10′-carotenal was diluted in a mixture of pulverized vitamin A-deficient, carotenoid-free diet and water (1:1, g: mL) to yield the aforementioned doses, assuming a daily intake of 4 g food.

### Liver mitochondria isolation

Animals were sacrificed by CO_2_ inhalation, and liver mitochondria were isolated^48^. Briefly, the liver was removed and immediately cooled at 4 °C in homogenization medium containing 0.32 M sucrose (J.T. Baker 4097–04), 1 mM EDTA (Sigma E5134) and 10 mM Tris-HCl (J.T. Baker X171–05). The tissues were washed and cut into small pieces. Then, 5 mL of the homogenization medium was added, and homogenization was performed in a glass Potter-Elvehjem homogenizer. Homogenates were centrifuged for 5 min at 1000 × *g* and 4 °C to pellet unbroken tissue, cells and nuclei. The supernatants were centrifuged for 10 min at 8,000 × *g* and 4 °C. The pellets were resuspended in 1 mL of buffer containing 10 mM Tris-HCl, 25 mM sucrose, 75 mM sorbitol (J.T. Baker V045-07), 100 mM KCl (J.T. Baker 3040-01), 10 mM K_2_HPO_4_, 0.05 mM EDTA and 5 mM MgCl_2_. A Bradford protein assay was performed to measure the mitochondrial protein concentration.

### HPLC and LC-MS analyses

Retinoid and/or β-carotene concentrations in embryos, maternal serum, liver and liver mitochondria were measured by reversed-phase HPLC as described previously^11,49^. Briefly, a whole embryo, 100 μL serum or ~100 μg liver was homogenized in 1–2 mL PBS, or 100 μL of isolated mitochondria were used. Internal standards (retinyl acetate for retinoid analysis, and echinenone for β-carotene analysis) were immediately added to 100–1000 μL of the homogenized sample. For retinoid extraction, samples were deproteinated by the addition of an equal volume of ethanol, then extracted with 4 mL hexanes by vortexing for 1–2 minutes. After centrifugation for 3 minutes at 1400 × *g*, the supernatants were collected and dried under N_2_ gas.

For carotenoid extraction, samples were sequentially mixed by vortexing with an equal volume of methanol, 0.4–0.9 mL acetone and 1 mL petroleum ether; the latter step was repeated once. After centrifugation for 3 minutes at 1400 × *g*, the supernatants were collected and dried under N_2_ gas. The evaporated residues were resuspended in 50 μL mobile phase (acetonitrile:methanol:dichloromethane, 70%:15%:15%) and injected into a 4.6 × 250 mm Ultrasphere C_18_ column (Beckman, Fullerton, CA) preceded by a C_18_ guard column at a flow rate of 1.8 mL/min.

Retinoids were detected at 325 nm, and β-carotene was detected at 450 nm. After integration and spectral analysis of the resulting peaks, retinoid and β-carotene concentrations were quantified with standard curves of AUC_compound_: AUC_standard_ vs. ng_compound_: ng_standard_. The limits of detection were <1 ng/dL and <10 ng/g for β-carotene, and <0.1 ng/dL and <1 ng/g for retinoids.

LC-MS analysis (detection limit: 1 ppm) was performed as described^26^.

### Synthesis of β-apo-10′-carotenal

β-apo-10′-carotenal was synthesized from ethyl β-apo-10′-carotenoate as described previously^50^.

### RNA extraction and qPCR analysis

RNA extraction and qRT-PCR analysis were performed as described previously^51^. Briefly, 50–200 mg of tissue was homogenized in TriPure (Roche 11667157001) isolation reagent (2 mL/100 mg tissue), incubated 5 min at room temperature, and mixed with chloroform (0.2 mL/mL TriPure) by vigorous shaking for 15 sec. After incubation on ice for 10 minutes, samples were centrifuged 15 min at 12,000 × *g*, and the supernatants were collected and mixed with isopropanol (0.5 mL/mL TriPure) by vortexing. Following 30 min incubation on ice and 15 min incubation at room temperature, samples were centrifuged 10 min at 12,000 × *g*, and the RNA pellet was washed twice with cold 75% diethyl pyrocarbonate (DEPC)-treated ethanol. The pellet was air-dried and re-suspended in DEPC water, and this RNA extract was stored at −80 °C for later analysis. The RNA concentration was determined on a Nanodrop Spectrophotometer, and RNA quality was assessed by the ratio of absorbances at 260 and 280 nm. RNA was DNAse-treated, and 1 μg was reverse-transcribed to cDNA according to the manufacturer’s instructions (Roche First-strand cDNA Synthesis kit, 04897030001).

qRT-PCR analysis was performed on duplicate/triplicate cDNA samples and controls containing no reverse transcriptase or no template, with SYBR Green Master Mix (Roche, 04887352001) in a Roche Light-cycler 480. The thermal cycling conditions were: 95 °C initiation for 10 minutes and 40–42 cycles of 95 °C for 30 sec, 58–60 °C annealing for 1 min, and 72 °C extension for 1 min. Relative gene expression was quantified by the comparative C_T_ method^52^, except for *Gpx4* (mitochondrial and total), which were analyzed by the standard curve method due to unequal amplification efficiencies between the gene of interest and the normalizer (β-*actin*). Normalized results are expressed as fold-changes from the control group, as indicated in the figure legends.

### Cellular viability

Cells were seeded in a 96-well plate (10,000 cells/well) in complete culture medium (DMEM [Cellgro #10–017], 10% fetal bovine serum, and 1% each of Glutamax [L-glutamate] and Penicillin/Streptomycin). The next day, cells were extensively washed with PBS and incubated for 24 hours with serum-free medium (DMEM, 0.05% bovine serum albumin, and 1% each of Glutamax [L-glutamate] and Penicillin/Streptomycin). Cells were then treated with β-carotene, β-apo-10′-carotenal, or Veh (<0.2% THF/methanol, <0.2% ethanol for β-apo-10′-carotenal) overnight. The following day, cells were then incubated with 10 μL WST-1 (Roche) for 0.5–2 hours to detect cellular proliferation. The absorbance was read in a microplate reader at 450 nm and 630 nm, and was corrected against a cell-free sample containing medium and the reagent.

### 8-oxoG ELISA

Total DNA was isolated from the uterus according to standard procedures. Equal amounts of total DNA from each sample within the same experimental group were pooled, and 10 μg of DNA of each pool was analyzed in duplicate with a commercially available 8-oxoG ELISA kit (Trevigen, Gaithersburg, MD) following the manufacturer’s procedures. Each DNA pool comprised DNA from the uteri of four *Bco2*^−/−^*Rbp4*^−/−^ virgin females on the regular chow diet, supplemented with the high dose of β-carotene or vehicle (as above) for three days and sacrificed 4 hours after the last injection.

### Statistical analysis

SPSS statistical software (IBM SPSS Statistics, version 16) was used for statistical analysis. The distribution of the data was determined by the Shapiro-Wilk test, and normally distributed data were analyzed by Student’s *t* test or analysis of variance (ANOVA) followed by *post hoc* analysis (Tamhane’s analysis for groups with unequal variance, or least significant difference [LSD] for groups with equal variance). Nonparametric data were analyzed by the Mann-Whitney U test, or the Kruskal-Wallis test followed by the Mann-Whitney U test for individual comparisons. Data are presented as the mean ± standard deviation (SD) or standard error of the mean (SEM), or the geometric mean and (range).

### Data analysis

All data generated or analyzed during this study are included in this published article (and its Supplementary Information file).

### Data availability

The datasets generated and analyzed during the current study are available from the corresponding author on reasonable request.

## Electronic supplementary material

Supplementary Information
