# Supplementary material for: β-apo-10′-carotenoids support normal embryonic development during vitamin A deficiency
Source: Sci Rep. 2018 Jun 11;8:8834. doi: 10.1038/s41598-018-27071-3 (PMC5995931; doi:10.1038/s41598-018-27071-3)
Supplement: Supplementary file 1 — Supplementary Information [file 41598_2018_27071_MOESM1_ESM.pdf]

Supplementary Information

β-apo-10'-carotenoids support normal embryonic development during vitamin A deficiency

Elizabeth Spiegler[1,#], Youn-Kyung Kim[1,#], Beatrice Hoyos[2], Sureshbabu Narayanasamy[3,4], Hongfeng Jiang[5], Nicole Savio[1], Robert W. Curley Jr.[3], Earl H. Harrison[4], Ulrich Hammerling[1,2], Loredana Quadro[1,*].

[1]Department of Food Science; Rutgers Center for Lipid Research; and New Jersey Institute for Food, Nutrition, and Health, Rutgers University, New Brunswick, New Jersey 08901, United States.

[2]Immunology Program, Memorial Sloan-Kettering Cancer Center, New York, New York, 10065, United States.

[3]College of Pharmacy, The Ohio State University, Columbus, OH 43210, United States.

[4]Department of Human Sciences, The Ohio State University, Columbus, OH 43210, United States.

[5]College of Physicians and Surgeons, Department of Medicine, Columbia University, New York, NY 10032, United States.

*Corresponding author. Email: lquadro@sebs.rutgers.edu (L.Q.)

[#]Both authors contributed equally to this work.

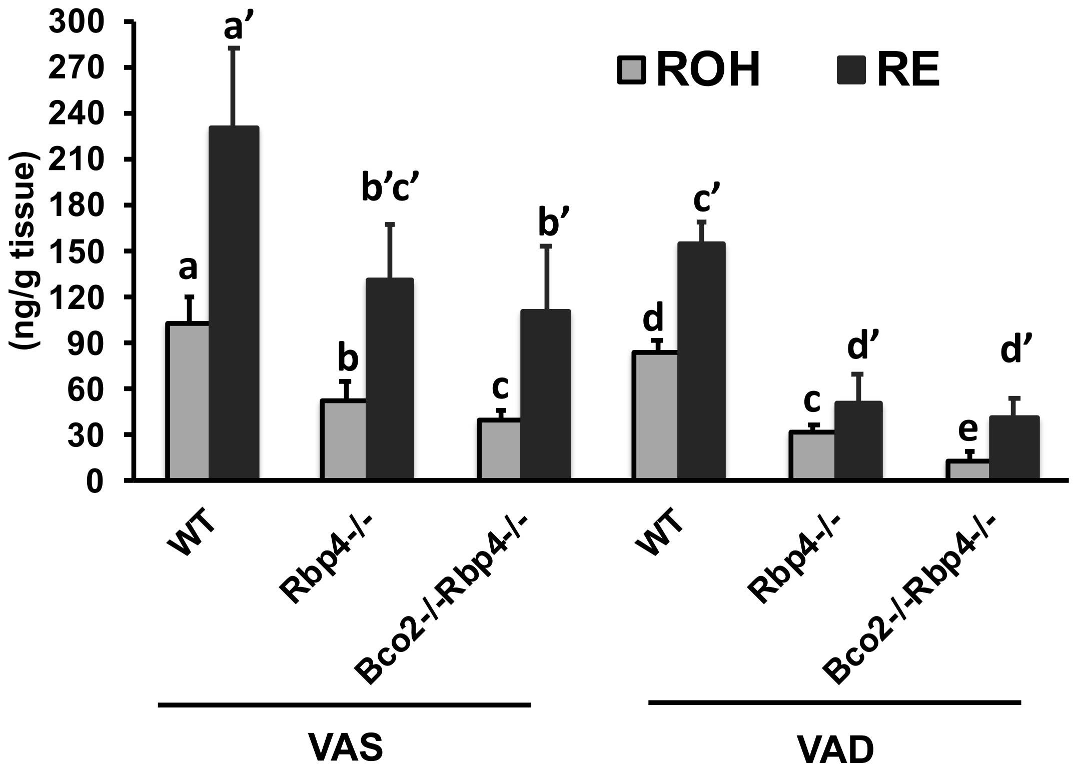

Figure S1. HPLC analysis of retinol (ROH) and retinyl esters (RE) in wild-type (WT), *Rbp4*$^{-/-}$ and *Bco2*$^{-/-}$*Rbp4*$^{-/-}$ 14.5-dpc embryos from dams of the same genotype fed a VAS or VAD diet from 0.5 dpc. n= 6-15 embryos/group. Data are means ± SDs. Labeled means (within each metabolite) without a common letter differ, $p<0.05$. The data for *Rbp4*$^{-/-}$ and *Bco2*$^{-/-}$*Rbp4*$^{-/-}$ embryos are the same as those shown in Fig. 1A.

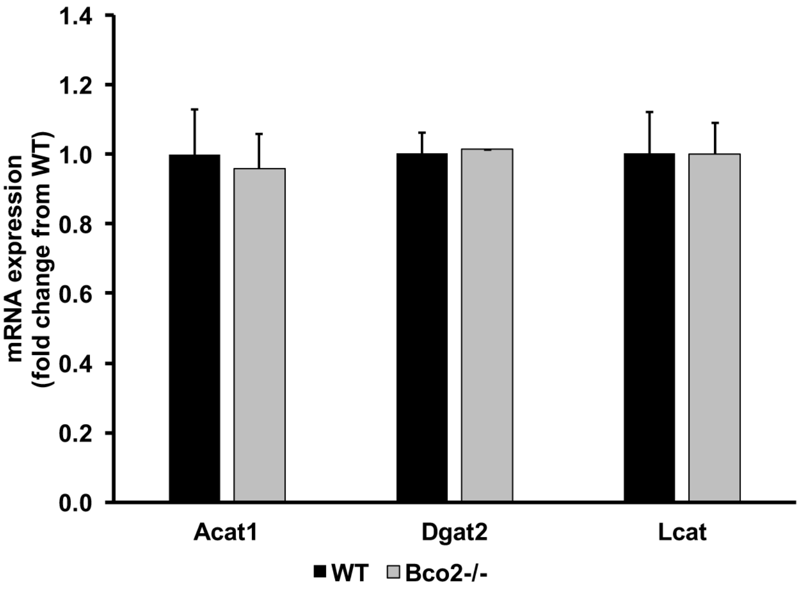

Figure S2. qPCR analysis of *Acat1, Dgat2* and *Lcat* expression in *Bco2*$^{-/-}$ and WT embryos at 14.5 days of gestation. WT embryos were set as the calibrator at 1. Data are means ± SDs fold of WT. For each embryonic genotype, 5 embryos (1-2 embryos/dam) were analyzed in triplicate, either individually or as pooled samples. Statistical analysis was performed by Student's *t* test to compare embryos from *Bco2*$^{-/-}$ vs. WT dams maintained on the regular chow diet. *, $p<0.05$ vs. WT.

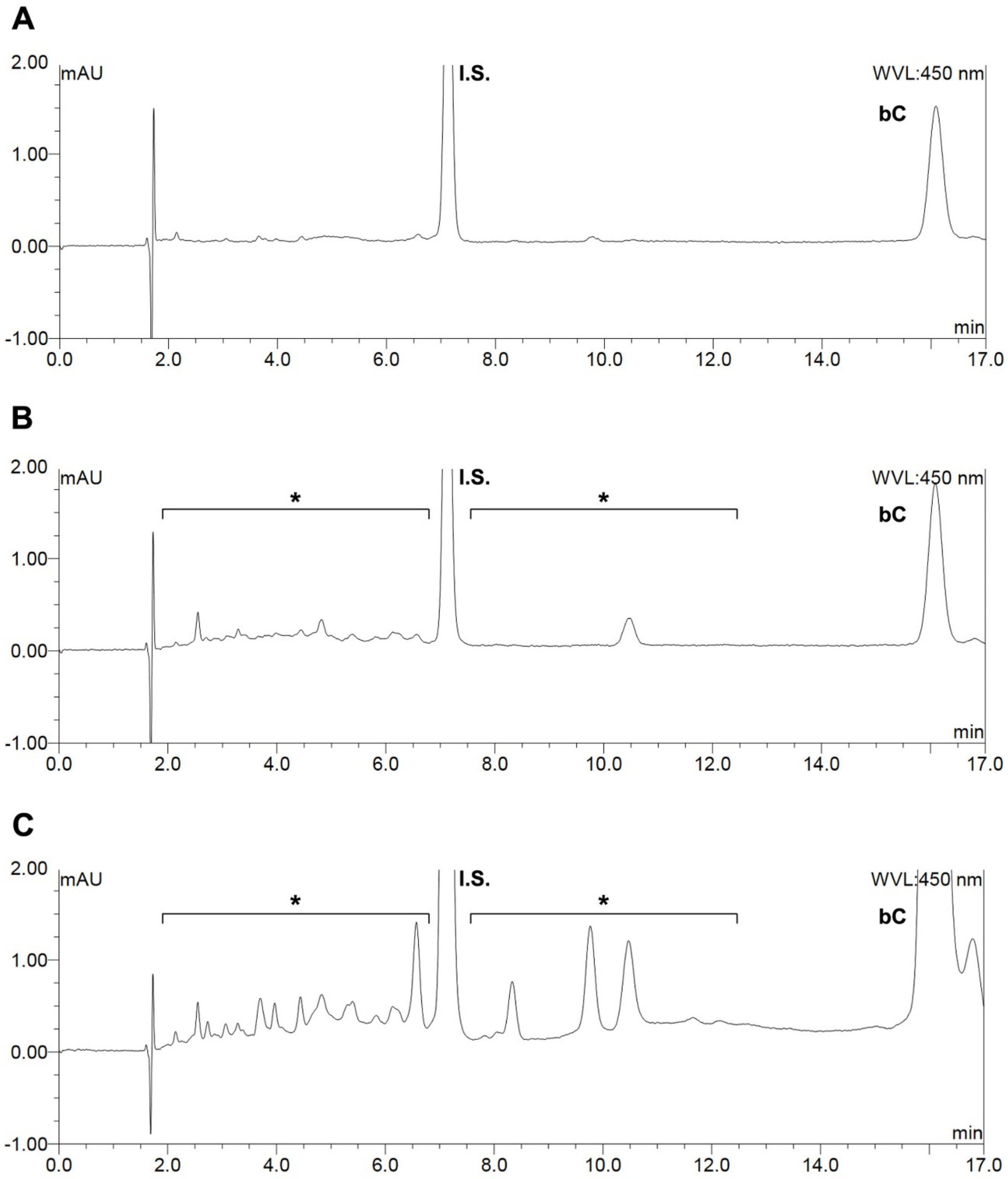

Figure S3. HPLC chromatograms of carotenoids extracted from a β-carotene solution, mouse serum and liver. Carotenoids were extracted from a β-carotene (bC) solution prepared for intraperitoneal injection (A), or from the serum (B) and liver (C) of a representative *Bco1*$^{-/-}$*Bco2*$^{-/-}$*Rbp4*$^{-/-}$ mouse injected with the bC solution. The extracts

were separated by HPLC, and the peaks in the resulting chromatograms were analyzed according to their retention times and absorbance spectra. Aside from bC and the Internal Standard (I.S., echinenone), peaks with absorbance maxima in the range of 404-452 nm were detected (indicated by *). Note the relatively small number of peaks in the chromatogram of the bC solution compared with serum and liver.

Table S1. Retinol and retinyl ester concentrations of WT and *Bco2$^{-/-}$* embryos

Genotype	n	Retinol (ng/g)	Retinyl Ester (ng/g)
		Vitamin A-sufficient diet	
WT	15	102 ± 17	230 ± 52
Bco2$^{-/-}$	12	107 ± 14	144 ± 33*
		Vitamin A-deficient diet	
WT	8	83 ± 8	154 ± 14
Bco2$^{-/-}$	8	84 ± 7	92 ± 21*

Dams were fed a vitamin A-sufficient chow diet (18 IU vitamin A/g and <1.2 μg/g β-carotene) or a purified vitamin A-deficient diet (< 0.2 IU/g and 0 μg/g β-carotene or other carotenoids) throughout pregnancy, and their embryos were collected at 14.5 dpc for HPLC analysis. Data are mean ± SD. Statistical analysis was performed within each dietary regimen by Student's *t* test or the Mann-Whitney U test as appropriate. *, p<0.05 vs. WT.
